# Transfer factors peptides (Imuno TF^®^) modulate the lung inflammation and airway remodeling in allergic asthma

**DOI:** 10.3389/fimmu.2022.1030252

**Published:** 2023-01-04

**Authors:** Carlos Rocha Oliveira, Jessica Carvalho, Fabiana Olímpio, Rodolfo Vieira, Flavio Aimbire, Hudson Polonini

**Affiliations:** ^1^ Medical School, Group of Phytocomplexes and Cell Signaling, Anhembi Morumbi University, São José dos Campos, São Paulo, Brazil; ^2^ Postgraduate Program in Biomedical Engineering, Anhembi Morumbi University, Sao Jose dos Campos, São Paulo, Brazil; ^3^ Department of Science and Technology, Federal University of Sao Paulo, Sao Jose dos Campos, São Paulo, Brazil; ^4^ Post-Graduate Program in Sciences of Human Movement and Rehabilitation, Federal University of Sao Paulo, Sao Jose dos Campos, Brazil; ^5^ Post-Graduate Program in Human Movement and Rehabilitation and in Pharmaceutical Sciences, Evangelical University of Goias (Unievangelica), Anapolis, Brazil; ^6^ Fagron (Netherlands), Rotterdam, Netherlands

**Keywords:** allergic asthma, airway remodeling, Th1/Th2/Treg cytokines, transfer factors peptides, lung inflammation

## Abstract

**Background:**

Allergic asthma is a chronic lung disease in which the lung inflammation and airway remodeling are orchestrated by both the inflammatory and the immune cells that creates a lung millieu that favors the perpetuation of clinical symptoms. The cell signaling in asthma involves the mast cells activation during initial contact with the allergen and, principally, the participation of eosinophils as well as Th2 cells which determine increased levels of IgE, exaggerated secretion of mucus and collagen, and bronchial hyperreactivity. Moreover, allergic asthma presents lower level of cytokines associated to the both Th1 and Treg cells response, and it implies in deficiency of anti-inflammatory response to counterregulate the exaggerated inflammation against allergen. Therefore, the equilibrium between cytokines as well as transcription factors associated to Th2, Th1, and Treg cells is compromised in allergic asthma. Imuno TF^®^ is a food supplement with ability to interfere in immune system pathways. It has been previously demonstrated that Imuno TF^®^ upregulated Th1 cell response whilst downregulated Th2 cell response in human lymphocytes.

**Objective:**

For this reason, we hypothesized that the Imuno TF effect could be restore the balance between Th1/Th2 CD4 T cells response in murine allergic asthma.

**Methods:**

Initially, animals were sensitized with OVA *via* i.p. and challenged with OVA i.n. on days 14, 15 and 16. Treatment with Imuno TF once a day was performed *via* orogastric from day 17 to day 20. Mice were euthanized on day 21.

**Results:**

The Imuno TF reduced eosinophilia, mucus production, and airway remodeling (collagen deposition) in asthma mice. Imuno TF influenced cellular signaling associated to allergic asthma once downregulated STAT6 expression as well as decreased IL-4, IL-5, and IL-13 in lung and serum. In addition, Imuno TF restored T-bet and Foxp3 expression as well as increased IL-12, IFN-ɣ, and IL-10.

**Conclusion:**

Ultimately, Imuno TF mitigated the allergic asthma due to the restoration of balance between the responses of Th1/Th2 as well as Treg cells, and their respective transcription factors the T-bet/STAT6 and Foxp3.

## 1 Introduction

Allergic asthma is an allergen-induced chronic disease in which lung inflammation (1), with massive presence of immune response cells and their pro-inflammatory mediators, is associated with airway remodeling, characterized by intense mucus production and exaggerated deposition. of collagen fibers in the lung parenchyma, and bronchial hyperreactivity, known as an exaggerated contractile response of the bronchial smooth muscles, are the main pulmonary alterations responsible for the decline in lung function in asthmatic individuals (2).

The cell signaling responsible for the symptoms of allergic asthma involves the participation of mast cells, eosinophils, and lymphocytes that orchestrate the immune and inflammatory response during the antigen recognition phase until the formation of a response associated with the activation of Th2 lymphocytes and secretion of pro-inflammatory mediators. IL-4, IL-5, and IL-13 (3). These cytokines associated with the Th2 response induce IgE immunoglobulin production by B lymphocytes (IL-4), chemotaxis for eosinophils towards the lung microenvironment (IL-5), mucus and collagen secretion, and bronchial hyperreactivity (IL-13) (4). In addition to Th2 cell activation, allergic asthma in mice as well as in asthmatic individuals also shows a significant reduction in the secretion of anti-inflammatory cytokines, such as IL-10 (5). In this sense, regulatory cells (Tregs) stand out because it secretes concentrations of IL-10 capable of counteracting the pro-inflammatory action of the Th2 response (6). Likewise, Th1 cells also show reduced secretion of IL-2, IL-12, and IFN-ɣ. These cytokines contribute to attenuating the Th2 response. Thus, there is an imbalance between the pro-inflammatory response associated to Th2 cells and the anti-inflammatory response mediated by Th1 and Treg cells (7).

The imbalance of cytokines secretion from CD4 T cells of Th2, Th1 and Treg subtypes is reflected in the transcription factors associated with these cytokines (8). It is well known that T-bet is presents in Th1 cells and it regulates the cell signaling that culminate in secretion of IL-2, IL-12, and IFN-ɣ (9). Conversely, STAT6 is a transcription factor present in Th2 cells with ability to induce secretion of IL-4, IL-5, and IL-13, which are responsible for the main features of asthma (10). However, murine models of allergic asthma as well as asthmatic individuals present high levels of STAT6 in airway, while both genic expression of T-bet and Foxp3 are drastically downregulated in lung tissue (11, 12).

Even today, the mortality rate is high and the efficiency of pharmacological therapy is limited in allergic asthma (13, 14). Thus, it is necessary to investigate promising anti-inflammatory therapies that have few adverse effects and can be combined with other therapies.

In this scenario, the natural-derived products or metabolites have always been used for diverse health applications, immunity strengthening included. In recent years, this has been gaining even more momentum, as pandemics caused by MERS and SARS-CoV-2 have aroused – and several natural compounds show antiviral activity and properties that help the organism keep the normal function of the immune system (15). Within this context, one such product is Imuno TF^®^, a nutritional supplement known by the scientific community as transfer factors (TF) due to the its effects on immune response (16).

Although immunological transfer factors are still controversial due to the lack of studies that can adequately address their characterization and functionality, our group has investigated such molecules from a specific food supplement to standardize the findings. In fact, our group has shown that oral feeding of Imuno TF^®^ is efficient and safe (17). Subsequently, our group demonstrated that Imuno TF^®^ upregulated Th1 cytokines and downregulated Th2 cytokines, once Imuno TF^®^ decreased secretion of IL-4, IL-5, IL-6, IL-13 and TNF, and conversely Imuno TF^®^ increased secretion of anti-inflammatory IL-10 (18). Both studies have helped us understand the action mechanism of Imuno TF^®^ on the immune system, which clinically was shown to improve the recovery of patients of COVID-19 (19). Therefore, it is reasonable to suggest that Imuno TF^®^ can help to control the imbalance of cytokine secretion associated with Th2/Th1/Treg responses in murine allergic asthma.

Thus, the Imuno TF^®^ effect on lung inflammation and airway remodeling in allergic asthma target secretion modulation of inflammatory cytokines associated to Th1, Th2 and Treg cells.

## 2 Methods

### 2.1 Animals

Two-month-old male C57Bl/6 mice were used. They were purchased from Center for the Development of Experimental Models (CEDEME) of the Federal University of São Paulo (UNIFESP), housed under controlled humidity, light and temperature conditions, inside ventilated polyethylene cages, in the vivarium located at Science and Technology Institute at the UNIFESP in São José dos Campos, SP, Brazil. The animals had food and water ad libitum. The mice were anesthetized with ketamine (100 mg/kg) and xylazine (10 mg/kg) *via* i.p. and euthanized with excess anesthetics. The experiments were approved according to CONCEA and the Research Ethics Committee on Animal Use of UNIFESP under the register 6844251018.

### 2.2 Murine model of allergic asthma and treatment with Imuno TF

Mice were initially sensitized on day 0 and seventh day with 100μg of ovalbumin (OVA) (grade V; Sigma-Aldrich, St. Louis, MO) in 100μL of sterile saline and adsorbed onto 50μL of inject aluminum hydroxide i.p (2.25 mg). Subsequently, mice were anesthetized with ketamine (200 mg/kg) and xylazine (10 mg/kg) intraperitoneally and challenged with 100μg of OVA in 50μL of sterile saline *via* intranasal on fourteenth, fifteenth and sixteenth day. Treatment with 0.1mg/Kg of Imuno TF was performed once a day by *via* orogastric (gavage) from seventeenth to twentieth day. Immuno TF^®^ is a nutritional supplement composed of oligo- and polypeptides fractions from porcine spleen commonly referred to as transfer factors. In according to Polonini and colleagues (20) previously described its composition. Mice were euthanized with excessive dose of anesthetic on twentieth-one day.

### 2.3 Experimental groups

**Figure 1:**
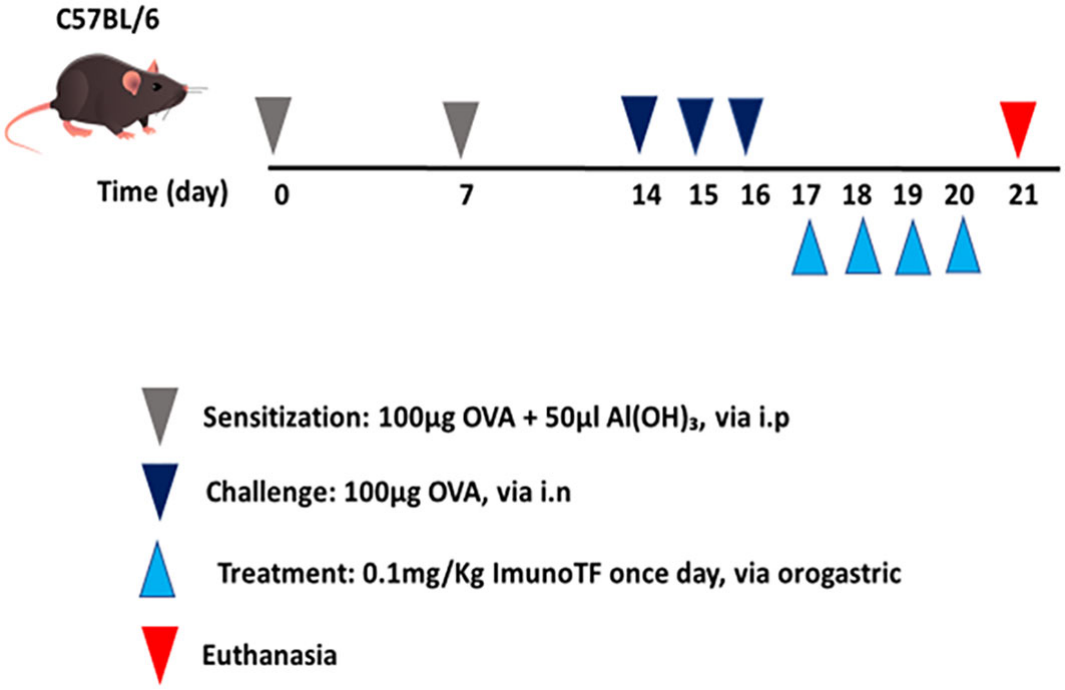
Schedule of allergic asthma model and treatment with Imuno TF. Mice were initially sensitized on day 0 and the seventh day with OVA. Subsequently, mice were challenged with OVA *via* intranasal on the fourteenth, fifteenth, and sixteenth days. Treatment with Imuno TF (0.1 mg/Kg) was performed once a day via orogastric (gavage) from the seventeenth to the twentieth day. Mice were euthanized with excessive doses of anesthetic on the twentieth-one day.

All experimental assays were performed in BALB/c mice which were divided in 4 groups, with a n=7 mice for each group, are them: (1) control – mice not manipulated; (2) ImunoTF – mice treated with ImunoTF; (3) asthma – mice challenged with OVA; (4) asthma + ImunoTF – mice challenged with OVA and treated with ImunoTF. The experimental design is illustrated in **Figure 1**.

### 2.4 Cellularity in bronchoalveolar lavage fluid

Total and differential cell counts of BALF fluid were determined by hemocytometer and cytospin preparation stained with Instant-Prov. Numbers of eosinophils, macrophages, neutrophils, and lymphocytes were scored by light microscopy.

### 2.5 Histology and morphometry

After the euthanasia, the lungs were carefully removed, perfused, and fixed with 10% paraformaldehyde for histological examination. Lung segments of approximately 5μm were stained with hematoxylin and eosin (Sigma-Aldrich Co.), Toluidine blue, Picrosirius, and Periodic Acid-Schiff (PAS). The parameters analyzed were peribronchial inflammation (polymorphonuclear cells), mast cell count, deposition of collagen fibers and deposition of mucus. Five airways of all animals were imaged at 400x magnifications using an Olympus BX 43 microscope camera and software CellSens Standard and Image Pro-Plus software (4.5, NIH, Maryland, EUA). The color threshold for Toluidine blue (mast cells), Picrosirius (collagen), and periodic acid Shiff (PAS) (mucus) were determined and the analyses were performed.

### 2.6 IgE level and cytokine measurement in serum

Serum was obtained from the blood sample. The total IgE as well as the cytokines IL-4, IL-5, IL-13, IL-2, IFN-ɣ, and IL-10 in serum were analyzed with ELISA specific kit (Invitrogen, SP, Brazil) according to the manufacturer’s instructions. To determine OVA-specific IgE levels OVA-specific IgE determination serum samples were added followed by the addition of biotin-labeled OVA (Sigma, SP, Brazil) according to the manufacturer’s instructions. Values were represented as ng/mL.

### 2.7 Lung homogenate and cytokine measurement

After euthanizing the animals, the lungs were removed and stored in a microtube at -80°C for later Elisa tests and qPCR. After thawing, 100 mg of lung tissue were placed in specific microtubes with ceramic beads and 1 mL of sterile PBS to perform the homogenate in the Precellys ^®^ equipment (Rockville, MD) through two cycles of 10 seconds at 68000rpm. After that, the samples were centrifuged at 5000 rpm, 4°C for 15 minutes, and the supernatant was collected and stored at -80°C for cytokines measurement. The concentration of IL-2, IL-4, IL-5, IL-13, IL-12, IFN-γ, and IL-10 in lung tissue were quantified using ELISA specific kits. Values were represented as pg/mL.

### 2.8 Real-time PCR for STAT6, T-bet, and Foxp3 in lung

For mRNA analysis, the thoracic cavity of the mice was exposed, and their heart and lung were removed in bloc, 24 hours after last OVA challenge. The pulmonary artery was cannulated and then the pulmonary vasculature was perfused with ice cold sterile phosphate buffer solution (PBS) using a peristaltic pump (Thermo Fisher Scientific, Suwannee, GA, USA) to remove the intravascular blood. Lung fragments were cut into 5 mm pieces using a tissue chopper, flash frozen in liquid nitrogen and stored at -80°C for real-time polymerase chain reaction (RT-PCR) analysis of genes expression. For that assay, total RNA was isolated from lung by TRIzol reagent (Gibco BRL, Gaithersburg, MD, USA) according to the manufacturer’s protocol. RNA was subjected to DNase I digestion, followed by reverse transcription to cDNA. PCR was performed in a 7000-sequence detection system (ABI Prism; Applied Biosystems, Foster City, CA, USA) using the SYBRGreen core reaction kit (Applied Biosystems). The STAT6 mRNA primers used for quantification were forward primer 5’- CCTGGTCGGTTCAGATGCTTT-3’ and reverse primer 5’- GTGCGGCAAGATGCTGTTTC-3’. For T-bet mRNA quantification were used forward primer 5’-GCCAGGGAACCGCTTATATG-3’ and reverse primer 5’-GACGATCATCTGGGTCACATTGT-3’. Primers used to quantify Foxp3 mRNA were forward primer 5’-CCCAGGAAAGACAGCAACCTT-3’ and reverse primer 5’-TTCTCACAACCAGGCCACTTG-3’. Primers for glyceraldehyde-3-phosphate dehydrogenase (GAPDH), forward 5′-CTCTACCCACGGCAAGTTCAA-3′ and reverse 5′-GGGATG ACCTTGCCCACAGC-3′, were used as control. Quantitative values for transcription factors and GAPDH mRNA transcription were obtained from the threshold cycle number, where the increase in the signal associated with an exponential growth of PCR products begins to be detected. Melting curves were generated at the end of every run to ensure product uniformity. The relative target gene expression level was normalized based on GAPDH expression as endogenous RNA control. ΔCt values of the samples were determined by subtracting the average Ct value of STAT6, T-bet, and foxp3 mRNA from the average Ct value of the internal control GAPDH. As it is uncommon to use ΔC_t_ as relative data due to this logarithmic characteristic, the 2^-ΔCt^ parameter was used to express the relative expression data. Results are expressed as a ratio relative to the sum of GAPDH transcript level as internal control.

### 2.9 Statistical analysis

The results were evaluated through the Analysis of Variance (ANOVA) and the Tukey-Kramer Multiple Comparison Test to determine the differences between the groups. The analysis was performed using Sigma Stat 3.1 software and graphs using GraphPad Prism 8.0 software. The results were considered significant when p < 0.05.

## 3 Results

### 3.1 Imuno TF attenuates inflammatory signals in lung and IgE level

**Figure 2:**
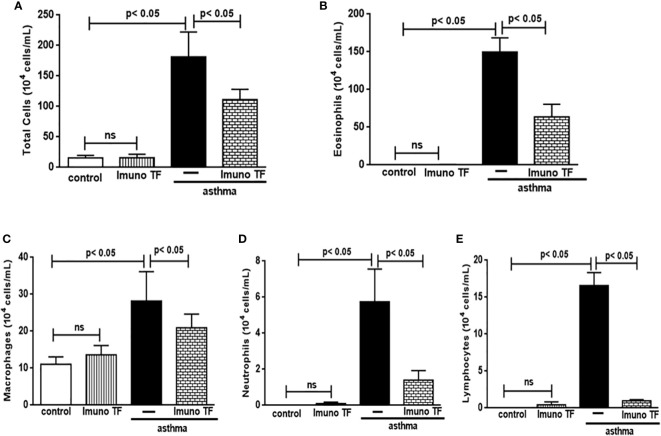
Imuno TF attenuates the cellularity in BALF. The total cells **(A)** and inflammatory cells were counted (x104) in BALF in millimeters by the morphometric evaluations of cytospin preparations. Treatment with Imuno TF (0.1 mg/Kg) was performed once a day via orogastric from the last challenge with OVA. The influx of specific leukocytes represented pulmonary inflammation; eosinophils **(B)**, macrophages **(C)**, neutrophils **(D)**, and lymphocytes **(E)** in BALF fluid. All cell counts were obtained from the control group, Imuno TF group, asthma group, and asthma + Imuno TF group. Each bar represents mean ± SEM from 7 different animals. Results were considered significant when p < 0.05. Non-significant results were labeled “ns”.

**Figure 3:**
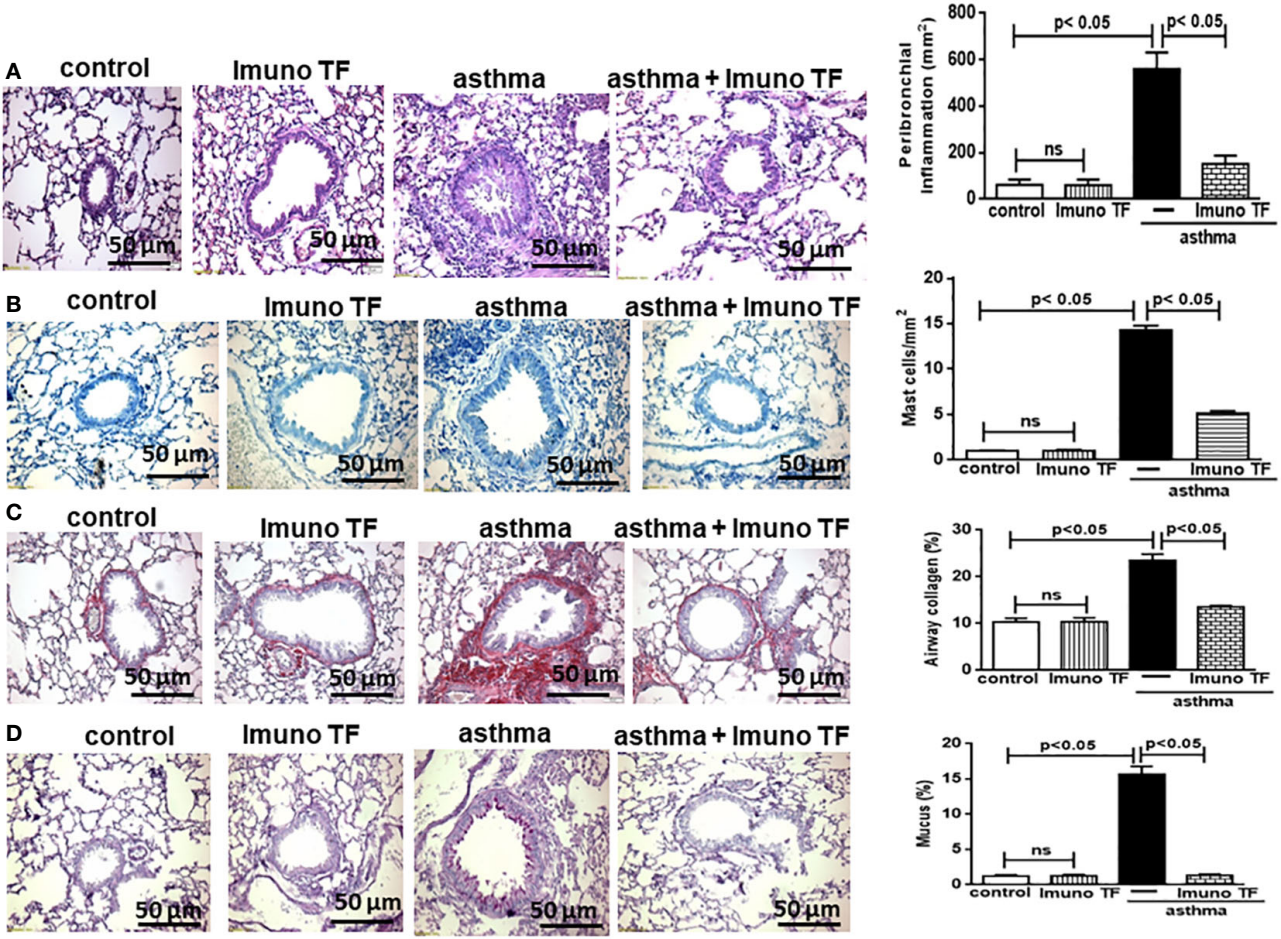
Imuno TF decreases the alterations in airway morphometry. Sections (5 μm) of formalin-fixed lungs were stained with hematoxylin and eosin for histological examination in control, Imuno TF, asthma, and asthma + Imuno TF groups. **(A)** Inflammation peribronchial. **(B)** Mast cells in the airway wall. **(C)** Deposition of collagen. **(D)** Deposition of mucus. Treatment with Imuno TF (0.1 mg/Kg) was performed once a day via orogastric from the last challenge with OVA. All quantifications were performed as described in the Material and Methods section. Each bar represents mean ± SEM from 7 different animals. Results were considered significant when p < 0.05. Non-significant results were labeled “ns”.

**Figure 4:**
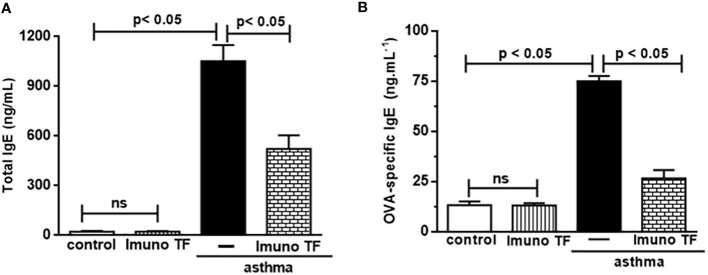
Imuno TF reduces the IgE levels in serum. Blood serum obtained from mice from control, ImunoTF, asthma, and asthma + Imuno TF was assayed for total IgE **(A)** and OVA-specific IgE **(B)** by using enzyme-linked immunosorbent assay (ELISA). OVA-specific IgE was determined by adding serum samples followed by biotin-labeled OVA (Sigma, SP, Brazil). Values were represented as ng/mL. Treatment with Imuno TF (0.1 mg/Kg) was performed once a day via orogastric from the last challenge with OVA. Each bar represents mean ± SEM from 7 different animals. Results were considered significant when p < 0.05. Non-significant results were labeled “ns”.

Infiltration of pro-inflammatory cells and airway remodeling are crucial factors in allergic asthma. The **Figure 2** illustrates the mice challenged with OVA (asthma group) presented total cells number (2A), eosinophils (2B), macrophages (2C), neutrophils (2D), and lymphocytes (2E) in BAL markedly higher than the control group. Otherwise, asthma mice pretreated with Imuno TF had a significant reduction of total cells number (2A), eosinophils (2B), macrophages (2C), neutrophils (2D), and lymphocytes (2E) in BAL compared to the asthma group. Whereas that Imuno TF attenuated inflammatory infiltrated in BAL of mice from asthma group, it seems reasonable to investigate if Imuno TF has ability to impair the inflammatory response in lung tissue. Therefore, we illustrated in **Figure 3** that peribronchial inflammation (3A), mast cells count (3B), deposition of collagen fibers (3C) and deposition of mucus (3D) was evidenced in the asthma compared to the control group. On the contrary, asthma mice pretreated with Imuno TF had a marked reduction of histological changes: peribronchial inflammation (3A), mast cells count (3B), deposition of collagen fibers (3C) and deposition of mucus (3D), compared to the asthma mice. Increased level of IgE is crucial for allergen recognition in allergic diseases, including asthma. Therefore, we investigated the Imuno TF effect on both total and OVA-specific IgE levels. The **Figure 4** illustrates levels of total (4A) IgE as well as OVA-specific IgE (4B) in serum of asthma mice higher than those found in mice from control group. Conversely, asthma mice pretreated with Imuno TF presented lower serum levels of total IgE (4A) and OVA-specific IgE (4B) compared to the asthma mice.

### 3.2 Imuno TF has dual effect on inflammatory cytokines in lung and serum

**Figure 5:**
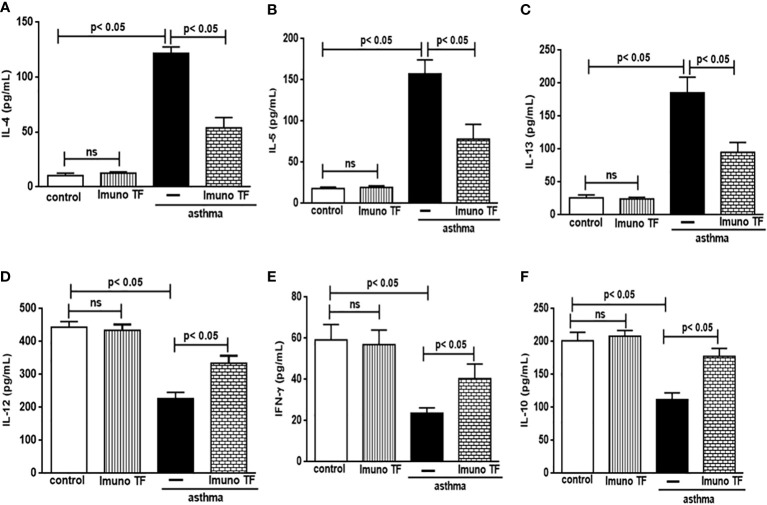
Imuno TF modulates the cytokines levels in the lung. Lung homogenate obtained from mice from control, Imuno TF, asthma, asthma + Imuno TF groups were prepared to analyze pro- and anti-inflammatory cytokines. Treatment with Imuno TF (0.1 mg/Kg) was performed once a day via orogastric from the last challenge with OVA. The cytokines IL-4 **(A)**, IL-5 **(B)**, IL-13 **(C)**, IL-12 **(D)**, IFN-γ **(E)**, and IL-10 **(F)** were assayed by enzyme-linked immunosorbent assay (ELISA). Each bar represents mean ± SEM from 7 different animals. Results were considered significant when p < 0.05. Non-significant results were labeled “ns”.

**Figure 6:**
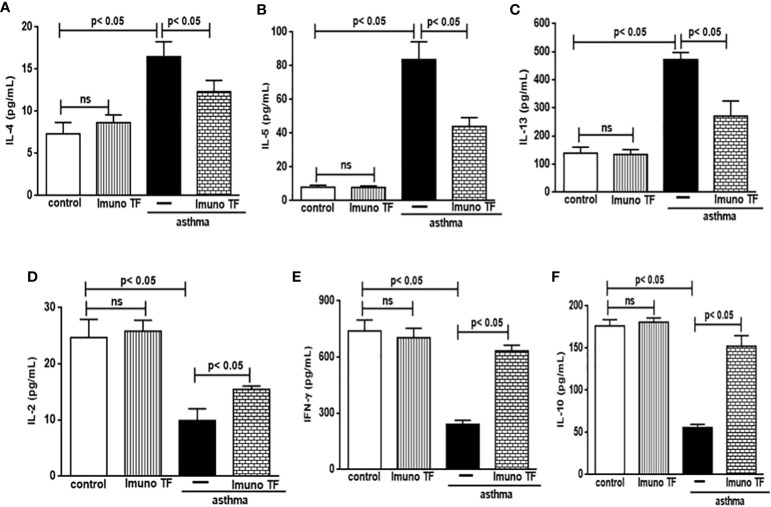
Imuno TF modulates the cytokines levels in serum. Serum obtained from control, Imuno TF, asthma, asthma + Imuno TF groups was prepared to analyze pro- and anti-inflammatory cytokines. IL-4 **(A)**, IL-5 **(B)**, IL-13 **(C)**, IL-2 **(D)**, INF-γ **(E)**, and IL-10 **(F)** were assayed by enzyme-linked immunosorbent assay (ELISA). Treatment with Imuno TF (0.1 mg/Kg) was performed once a day via orogastric (gavage) from the last challenge with OVA. Each bar represents mean ± SEM from 7 different animals. Results were considered significant when p < 0.05. Non-significant results were labeled “ns”.

In allergic asthma, the equilibrium between the Th2 response-associated pro-inflammatory cytokines and the anti-inflammatory cytokines linked to both the Th1 and Treg responses is compromised. For this reason, we investigate the immunomodulatory effect of Imuno TF on these cytokines in lung tissue and serum. Thus, the **Figure 5** illustrates a marked increase of pro-inflammatory cytokines associated to Th2 cells, are them: IL-4 (5A), IL-5 (5B), and IL-13 (5C), in lung tissue from asthma mice compared to the control group. Otherwise, asthma mice pretreated with Imuno TF presented reduced concentrations of IL-4 (5A), IL-5 (5B), and IL-13 (5C) in lung tissue compared to asthma mice. with Regard to anti-Th2 cytokines, the **Figures 5D–F** illustrate a significant fall of cytokines concentration IL-12, IFN-γ, and IL-10, respectively, in lung from asthma mice compared to the control group. On the contrary, the Imuno TF restored levels of IL-12 (5D), IFN-ɣ (5E), and IL-10 (5F) in lung of asthma mice. In order to investigate a systemic immunomodulatory action of Imuno TF, we illustrated in **Figure 6** the anti-Th2 effect of Imuno TF on IL-4 (6A), IL-5 (6B), and IL-13 (6C) in serum of asthma mice. These findings are relevant because the serum of asthma mice presented increased levels of these cytokines compared to the control group. Otherwise, the **Figure 6** shows also the pro-Th1 and pro-Treg effect of Imuno TF on IL-2 (6D), IFN-ɣ (6E), and IL-10 (6F) in serum of asthma mice. It is an important result, once levels of IL-2 (6D), IFN-ɣ (6E), and IL-10 (6F) in serum of asthma mice were higher than those found in the control group.

### 3.3 Imuno TF modulates gene expression of STAT6, T-bet, and Foxp3 in lung

**Figure 7:**
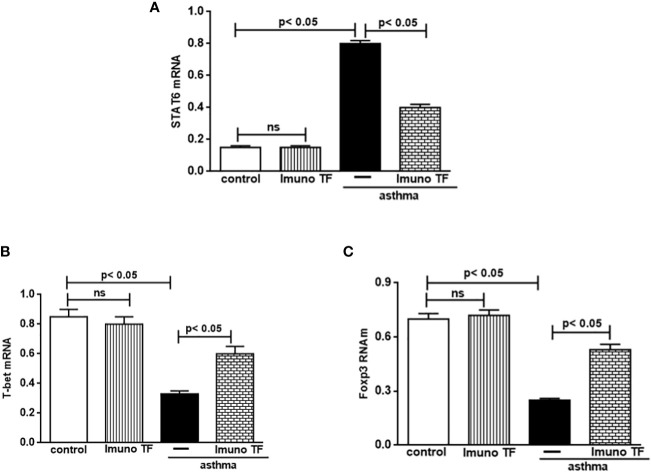
Imuno TF modulates the allergic asthma-associated transcription factors expression in the lung. The mRNA expression of the STAT6, T-bet, and Foxp3 was performed in the lungs of mice from the control, Imuno TF, asthma, asthma + Imuno TF groups. The mRNA expression for STAT6 **(A)**, T-bet **(B)**, and foxp3 **(C)** in lung tissue was evaluated through Real Time-PCR. Treatment with Imuno TF (0.1 mg/Kg) was performed once a day via orogastric (gavage) from the last challenge with OVA. Each bar represents mean ± SEM from 7 different animals. Results were considered significant when p < 0.05. Non-significant results were labeled “ns”.

The unbalance between pro-inflammatory and anti-inflammatory cytokines associated to Th2/Th1/Treg cells is also closely linked gene expression for STAT6, T-bet, and Foxp3 in allergic asthma. Whereas Imuno TF reduced IL-4, IL-5, and IL-13 in lung and serum of asthma mice, we tested if this agent could modulate the gene expression of allergic asthma-associated transcription factors. Thus, initially, the **Figure 7** illustrates a higher level of mRNA for STAT6 (7A) from asthma mice in comparison with control group. At the same time, there is a marked fall of gene expression for T-bet (7B) and Foxp3 (7C) in asthma group compared to the control group. Conversely, mice treated with Imuno TF and challenged with OVA had a significant reduction of gene expression for STAT6 (7A) compared to asthma mice. Likewise, the Imuno TF also influenced gene expression of T-bet and Foxp3 once asthma mice treated with Imuno TF presented restoration, even if partially, of gene expression for T-bet (7A) and Foxp3 (7B) compared to asthma group.

## 4 Discussion

Still currently, the most used pharmacological treatments to control chronic inflammation in asthma is steroids (21). Generally, asthma individuals use bronchodilators to allergic crisis and steroid to long term control the airway inflammation (22). However, asthmatic need to be treated chronically because the allergic asthma is chronic disease, and it is a limiting factor once it implies in diverse side effects which became the allergic asthma one of the main chronic lung diseases with high cost of treatment and hospitalizations that burden the public health system (23). In this scenario, allergic asthma urgently lacks therapies capable of attenuating lung inflammation and airway remodeling *via* modulation of cytokine secretion associated with subpopulations of Th2, Th1, and Treg cells.

Regarding to murine model of allergic asthma, it is known that any experimental model that proposes to induce an inflammatory disease cannot reliably reproduce the variations in symptoms that occur in humans. However, the value of experimental models is that they can reproduce the cellular signaling of the immune and inflammatory response that characterizes the disease. With the models of acute or chronic lung inflammation it is no different. Therefore, the experimental model of allergic asthma adopted in the present study also has limitations regarding the variation of symptoms. However, the main features of allergic asthma were reproduced, such as increased mast cell population, elevated IgE levels, pulmonary eosinophilia, exacerbated mucus secretion and exaggerated collagen deposition, and pro-inflammatory cytokine storm associated with Th2 response accompanied by failure in cytokine secretion associated with the Th1 response. Thus, it is reasonable to infer that the effect of Immuno TF was tested in a lung microenvironment with the same cell signaling observed in allergic asthma.

In this scenario, we performed experimental assays to teste whether ImunoTF, a kind of transfer factors peptides with immunomodulatory, is effective in allergic asthma. Our results show first time that this agent modulates lung inflammation and airway remodeling in murine asthma by modulating of cytokine secretion and transcription factors associated to subpopulations of Th2, Th1, and Treg cells.

Initially, our results show that after intranasal Imuno TF the inflammatory cells migration into bronchi of asthma mice, such as macrophages, neutrophils, with emphasis on the population of eosinophils and lymphocytes in the BALF were attenuated. In addition, it is well known that at the moment binding of mast cell antibody to allergen, occurs an immediate releasing of inflammatory mediators, including the chemotactic factor for eosinophils, which amplifies the allergic response in lung (24). The present study showed also that Imuno TF reduced the mast cells population in lung tissue of asthma mice. This finding deserves highlight since that the mast cell is critical in antigen recognition phase, and express high-affinity receptors for IgE (25). Likewise, Imuno TF reduced IgE concentration in serum of asthma mice. Taken together, the Immuno TF effect on mast cell population as well as IgE level indicates that this agent can modulates the initial phase of antigen-recognition.

In allergic asthma, the lymphocytes migration towards lung is accompanied by a massive releasing of pro-inflammatory mediators in lung microenvironmental with subsequent tissue damage (26). The results presented herein show that Imuno TF attenuated the lung tissue damage once it decreased inflammatory cells population, collagen deposition and mucus secretion in peribronchial area of asthma mice.

Individuals with allergic asthma, at different levels of disease severity, show an imbalance in the secretion of inflammatory cytokines linked to Th2, Th1, and Treg responses (27). In according to these authors, the findings in the present manuscript evidenced also an unbalance on Th2, Th1, and Treg responses in OVA-challenged mice. In fact, the level of Th1 cytokines IL-12 and IFN-ɣ as well level of Treg cytokine IL-10 in lung of asthma mice was lower than control, and inversely, Th2 cells response-associated the cytokines IL-4, IL-5, and IL-13 had a significant increase in lung of asthma mice compared to the control group. In this situation, the anti-inflammatory effect of both Th1 and Treg cytokines is suppressed by intense releasing of pro-inflammatory mediators from Th2 cells that trigger the chronicity of allergic asthma.

According to these findings, our results show that unbalance between Th cytokines was also present in serum of asthma mice. Indeed, both the cytokines IL-4 and IL-5 as well as IL-13 were augmented in asthma mice, while the level of anti-inflammatory cytokines IL-2, IFN-ɣ, and IL-10 were drastically reduced. Otherwise, our results demonstrated that the treatment with Imuno TF induced a marked fall of Th2 cytokines, whilst concomitantly induced high levels of Treg cytokine as well as Th1 cytokines in serum of asthma mice. Therefore, the present study shows first time that the Imuno TF effect is driven to modulation of Th cells response in allergic asthma. It is noteworthy that Immuno TF also modulated the secretion of serum cytokines, strengthening the idea that this transfer factor peptide to negatively modulates the immune response unbalance in allergic asthma.

The secretion of pro- and anti-inflammatory mediators from different subpopulations of Th cells depends on cell signaling orchestrated with transcription factors (28). It is well known that the transcription factor STAT6 play essential role in the allergic inflammatory response once are producers of Th2 cytokines IL-4, IL-5, and IL-13 which are responsible for maintaining IgE synthesis, mucus secretion and collagen deposition, causing exaggerated remodeling of the airway and limiting airflow (29–31). In accordance with these findings, our results herein show that Imuno TF negatively regulated gene expression for STAT6 in lung from allergic mice. Based on these data, we can infer that the anti-Th2 cytokine effect of Immuno TF is due to its downregulatory action on gene expression for STAT6. The unbalance of Th1/Th2 cells response in allergic asthma shows that the IL-4, IL-5, and IL-13 and its respective transcription factor STAT6 remains intensely upregulated at the expense of a drastic decrease of T-bet levels in the airway of individuals with allergic asthma (28). It is important highlights that T-bet depress STAT6 signaling (32). Our results show that Imuno TF upregulated the T-bet expression in lung of asthma mice, which was reflected in restoration of levels IL-12 and IFN-ɣ in lung to values close to the control group.

In addition to Th1 and Th2 responses, the interventions that restore the Treg cells response may be also useful for allergic asthma treatment. It is well known that Tregs cells have a crucial role in suppression of exaggerated immune response, once transcription factor Foxp3 is intimately linked with depresses function of Treg cells *via* secretion of IL-10 (33). Herein, the asthma mice presented drastic reduction of IL-10 secretion accompanied of lower expression of Foxp3 in lung. In contrast, our findings show that the Imuno TF upregulated the Foxp3 expression associated to increase of IL-10 secretion in lung of asthma animals, indicating that Imuno TF also upregulates Treg cells response.

Whereas that Immuno TF^®^ presented beneficial effect on murine model of allergic asthma, and that allergic asthma is a chronic disease, is important discuss if this agent produces side-effects. In according to Polonini and colleagues (20), the safety assessment of Immuno TF^®^ did not show mutagenic potential. In addition, the authors presented acute oral toxicity results, showing that Immuno TF^®^ at a dose of 2000 mg/kg can be classified as GHS Category 5 (“not classified” according to the Globally Harmonized System of Classification and Chemicals Labeling, GHS), with a cut-off value of LD50 in Wistar rats of 5000 mg/kg. As the recommended oral dose of Immuno TF^®^ ranges from 50 to 100 mg daily (equivalent to 0.7 to 1.4 mg/kg for an adult weighing 70 kg), we can infer that Immuno TF^®^ is safe for use at the recommended dose.

Taken together, our studies indicate that Imuno TF mitigated lung inflammation and airway remodeling by modulating on balance between the responses of Th2/Th1 cells as well as Treg cells and their respective transcription factors STAT6/T-bet and Foxp3.

## Data availability statement

The datasets presented in this study can be found in online repositories. The names of the repository/repositories and accession number(s) can be found in the article/supplementary material.

## Ethics statement

The animal study was reviewed and approved by the Ethics Committee on Animal Research from the Federal University of São Paulo at protocol number 6844251018.

## Author contributions

Conceptualization and Methodology, CO and FA. Investigation, JC and FO. Writing - Original Draft, CO and FA. Writing - Review and Editing, FA, CO, RV, and HP. Supervision, FA and CO. Funding Acquisition, CO and HP. All authors read and approved the final manuscript.

## References

[1] Holgate ST . The epidemic of allergy and asthma. Nature (1999) 402:B2–4. doi: 10.1038/35037000 10586888

[2] Maslan J Mims JW . What is asthma? Pathophysiology, demographics, and health care costs. Otolaryngologic Clinics North America (2014) 4:13–22. doi: 10.1016/j.otc.2013.09.010 24286675

[3] Holgate ST . Innate and adaptive immune responses in asthma. Nat Med (2012) 18:673–83. doi: 10.1038/nm.2731 22561831

[4] Hogg J . Pathology of asthma. J Allergy Clin Immunol (1993) 9:1–5. doi: 10.1016/0091-6749(93)90029-f 8335845

[5] Minnicozzi M Sawyer RT Fenton MJ . Innate immunity in allergic disease. Immunol Rev (2011) 242:106–27. doi: 10.1111/j.1600-065X.2011.01025.x 21682741

[6] Robinson DS . The role of the T cell in asthma. J Allergy Clin Immunol (2010) 126:1081–91. doi: 10.1016/j.jaci.2010.06.025 20709383

[7] Muehling LM Lawrence MG Woodfolk JA . Pathogenic CD4+ T cells in patients with asthma. J Allergy Clin Immunol (2017) 140:1523–40. doi: 10.1016/j.jaci.2017.02.025 PMC5651193 28442213

[8] Walford HH Doherty TA . STAT6 and lung inflammation. JAKSTAT (2013) 2:e25301. doi: 10.4161/jkst.25301 24416647 PMC3876430

[9] Anatriello E Cunha M Nogueira J Carvalho JL Sá AK Miranda M . Oral feeding of lactobacillus bulgaricus N45.10 inhibits the lung inflammation and airway remodeling in murine allergic asthma: Relevance to the Th1/Th2 cytokines and STAT6/T-bet. Cell Immunol (2019) 314:103928. doi: 10.1016/j.cellimm.2019.103928 31178059

[10] Krishnamurthy P Kaplan MH . STAT6 and PARP family members in the development of T cell-dependent allergic inflammation. Immune Netw (2016) 16:201–10. doi: 10.4110/in.2016.16.4.201 PMC5002446 27574499

[11] Kiwamoto T Ishii Y Morishima Y Yoh K Maeda A Ishizaki K . Transcription factors T-bet and GATA-3 regulate development of airway remodelling. Am J Respir Crit Care Med (2006) 174:142–51. doi: 10.1164/rccm.200601-079oc 16614350

[12] Bowen H Kelly A Lee T Lavender P . Control of cytokine gene transcription in Th1 and Th2 cells. Clin Exp Allergy (2008) 38:1422–31. doi: 10.1111/j.1365-2222.2008.03067.x 18647314

[13] Walsh GM . Emerging drugs for asthma. Expert Opin Emerging Drugs (2008) 4:643–53. doi: 10.1517/14728210802591378 19046132

[14] Blake KV . Drug treatment of airway inflammation in asthma. Pharmacotherapy (2001) 21:3S–20S. doi: 10.1592/phco.21.4.3s.34265 11253868

[15] Othman SI Nayel MA Alwaele MA al Fassam H Abu-Taweel GM Altoom NG . Immunology and controlling of coronaviruses; the current enemy for humanity: A review. Int J Biol Macromolecules (2021) 193:1532–40. doi: 10.1016/j.ijbiomac.2021.10.216 PMC8557928 34732305

[16] Ferreira AO Polonini HC Dijkers ECF . Postulated adjuvant therapeutic strategies for COVID-19. J 215 Personalized Med (2020) 10:80. doi: 10.3390/jpm10030080 PMC7565841 32764275

[17] Oliveira CR Vieira RP Ferreira AO Gonçalves AESS Polonini H . Immunoregulatory effects of imuno TF® (transfer factors) on Th1/Th2/Th17/Treg cytokines. J Clin Exp Immunol (2021) 6:421–31. doi: 10.33140/jcei.06.04.01

[18] Hernández MD Urrea J Bascoy L . Evolution of COVID-19 patients treated with a combination of nutraceuticals to reduce symptomatology and improve prognosis: A multi-centred, retrospective cohort study. J Clin Rev Case Rep (2021) 6:662–701. doi: 10.21203/rs.3.rs-133532/v2

[19] Steele RW Myers MG Vincent MM . Transfer factor for the prevention of varicella-zoster infection in childhood leukemia. New Engl J Med (1980) 303:355–9. doi: 10.1056/NEJM198008143030702 6248780

[20] Polonini H Gonçalves AESS Dijkers E Ferreira. AO . Characterization and safety profile of transfer factors peptides, a nutritional supplement for immune system regulation. Biomolecules (2021) 11:665–7. doi: 10.3390/biom11050665 PMC8145720 33947143

[21] Barnes PJ . Glucocorticoids. Chem Immunol Allergy (2014) 100:311–6. doi: 10.1159/000359984 24925411

[22] Barnes PJ . Molecular mechanisms of corticosteroids in allergic diseases. Allergy (2001) 56:928–36. doi: 10.1034/j.1398-9995.2001.00001.x 11576070

[23] Méndez-Enríquez E Hallgren J . Mast cells and their progenitors in allergic asthma. Front Immunol (2019) 29:821. doi: 10.3389/fimmu.2019.00821 PMC6548814 31191511

[24] Sastre B Rodrigo-Muñoz JM Garcia-Sanchez DA Cañas JA Del Pozo. V . Eosinophils: Old players in a new game. J Investigational Allergology Clin (2018) 28:289–304. doi: 10.18176/jiaci.0295 30059011

[25] Lambrecht BN Hammad H Fahy JV . The cytokines of asthma. Immunity (2019) 50:975–91. doi: 10.1016/j.immuni.2019.03.018 30995510

[26] Zhang H Kong H Zeng Z Guo L Sun X He S . Subsets of regulatory T cells and their roles in allergy. J Transl Med (2014) 12:125. doi: 10.1186/1479-5876-12-125 24886492 PMC4023533

[27] Finotto S Glimcher L . T Cell directives for transcriptional regulation in asthma. Springer Semin Immunopathol (2004) 25:281–94. doi: 10.1007/s00281-003-0143-1 15007632

[28] Hoshino A Tsuji T Matsuzaki J Jinushi T Ashino S Teramura T . STAT6-mediated signaling in Th2-dependent allergic asthma: critical role for the development of eosinophilia, airway hyper-responsiveness and mucus hypersecretion, distinct from its role in Th2 differentiation. Int Immunol (2004) 16:1497–505. doi: 10.1093/intimm/dxh151 15351784

[29] De Grove KC Provoost S Hendriks RW McKenzie AN Seys LJ Kumar S . Dysregulation of type 2 innate lymphoid cells and TH2 cells impairs pollutant-induced allergic airway responses. J Allergy Clin Immunol (2016) 16:30271–8. doi: 10.1016/j.jaci.2016.03.044 PMC5420012 27315767

[30] Tomita K Caramori G Ito K Sano H Lim S Oates T . STAT6 expression in T cells, alveolar macrophages and bronchial biopsies of normal and asthmatic subjects. J Inflammation-London (2012) 9:5. doi: 10.1186/1476-9255-9-5 PMC3364916 22401596

[31] Bellinghausen I Klostermann B Knop J Saloga J . Human CD4+CD25+ T cells derived from the majority of atopic donors are able to suppress TH1 and TH2 cytokine production. J Allergy Clin Immunol (2003) 111:862–8. doi: 10.1067/mai.2003.1412 12704370

[32] Szabo SJ Kim ST Costa GL Zhang X Fathman CG Glimcher. LH . A novel transcription factor, T-bet, directs Th1 lineage commitment. Cell (2000) 100:655–69. doi: 10.1016/s0092-8674(00)80702-3 10761931

[33] Lloyd CM Hawrylowicz CM . Regulatory T cells in asthma. Immunity (2009) 31:438–49. doi: 10.1016/j.immuni.2009.08.007 PMC3385348 19766086

